# Single-stage open window thoracostomy with simultaneous muscle flap transposition and early negative pressure wound therapy for chronic empyema: a propensity score- and machine learning-based study

**DOI:** 10.1186/s13019-026-03997-y

**Published:** 2026-04-03

**Authors:** Daiki Hayashi, Kensuke Kojima, Toshiteru Tokunaga, Kyoichi Okishio, Hyungeun Yoon

**Affiliations:** 1 Department of General Thoracic Surgery, NHO Kinki Chuo Chest Medical Center, 1180 Nagasone-cho, Kita-ku, Sakai-shi, 591-8555 Osaka Japan; 2 Clinical Research Center, NHO Kinki Chuo Chest Medical Center, Osaka, Japan; 3 Department of Thoracic Oncology, NHO Kinki Chuo Chest Medical Center, Osaka, Japan

**Keywords:** Bronchopleural fistula, Chronic empyema, Muscle flap transposition, Negative pressure wound therapy, Open window thoracostomy

## Abstract

**Background:**

To compare the clinical efficacy of a single-stage surgical approach combining open window thoracostomy, muscle flap transposition, and early negative pressure wound therapy (NPWT) with that of conventional staged management for chronic empyema.

**Methods:**

This retrospective, single-center cohort included 45 patients with chronic empyema (single-stage, *n* = 7; conventional, *n* = 38) who were treated from 2009 to 2024. The primary endpoint was the cavity reduction ratio measured via three-dimensional computed tomography (3D CT). To minimize selection bias, we applied propensity score matching (PSM) and inverse probability of treatment weighting (IPTW). We prespecified the average treatment effect on the treated (ATT) as the primary estimand, whereas the average treatment effect (ATE) was considered exploratory because treatment assignment was strongly related to bronchopleural fistula (all single-stage patients had BPF) and overlap was limited. The follow-up intervals were linearly normalized to adjust for time-dependent variability. Machine learning models were used as complementary predictive analyses to identify predictors of cavity reduction.

**Results:**

After PSM (*n* = 5 per group), the single-stage group had significantly greater cavity reduction than the conventional group did (90% [89–94%] vs. 37% [32–43%], *p* = 0.008). The IPTW analysis demonstrated significant treatment effects: 0.38 (95% CI: 0.17–0.59, *p* = 0.005) for the ATT and 0.63 (95% CI: 0.12–1.14, *p* = 0.03) for the ATE (exploratory). Machine learning models consistently identified the treatment approach and time-related variables as important predictors of cavity reduction.

**Conclusions:**

Compared with conventional management, the single-stage approach was associated with significantly greater empyema cavity reduction and was feasible even in patients with bronchopleural fistulas. Causal interpretation is most defensible for the ATT, whereas generalization to the full population (ATE) should be considered exploratory due to limited overlap. Despite the small sample size, the consistent findings across diverse analytical frameworks support the single-stage strategy as a promising approach that warrants prospective multicenter validation and clearer criteria for patient selection.

**Supplementary Information:**

The online version contains supplementary material available at 10.1186/s13019-026-03997-y.

## Background

Chronic empyema is a progressive pleural infection characterized by inflammation, loculated effusions, and fibrous septation. Inadequate treatment can result in prolonged hospitalization and recurrent infection [1]. Conventional management employs open window thoracostomy (OWT), which often relies on extended natural healing [2]. Residual cavities increase the risk of infection, highlighting the need for effective cavity reduction [3]. Muscle flap transposition and negative pressure wound therapy (NPWT) promote cavity obliteration and tissue granulation, respectively [4, 5]. Although NPWT has been used post-OWT or with delayed flap transposition, evidence is limited to case reports [6,7]. The efficacy of combining OWT, simultaneous muscle flap transposition, and early NPWT remains unevaluated. This study evaluated a single-stage strategy via propensity score matching and machine learning analyses. By concurrently addressing infection control and cavity reduction, this approach may shorten treatment and facilitate earlier reintegration. The study design and outcomes are summarized in Additional File 1: Figure S1.

## Methods

### Study population

This retrospective study included 45 patients who underwent OWT for chronic empyema between 2009 and 2024. Chronic empyema was defined as empyema persisting for > 4 weeks, with pleural thickening, loculated fluid, fibrosis, and restricted lung expansion [8, 9]. Patients were divided into conventional (*n* = 38: OWT with/without delayed NPWT or muscle flap) and single-stage (*n* = 7: simultaneous OWT, muscle flap, and NPWT within 72 h) groups (Additional File 1: Figure S2). Muscle flaps were pedicled; harvested from the intercostal, serratus anterior, or latissimus dorsi muscles; and used to fill the cavity and reinforce bronchopleural fistula (BPF). NPWT (V.A.C.^®^ Therapy [VAC], KCI, San Antonio, TX, USA) was initiated at − 50 to − 75 mmHg, increased to − 175 to − 200 mmHg, and continued for up to six weeks under national insurance coverage. The baseline characteristics included demographics, comorbidities, and surgical history. The primary outcome was cavity volume reduction, which was calculated as (preoperative − postoperative volume)/preoperative volume via 3D CT analysis (SYNAPSE VINCENT, Fujifilm Corporation, Tokyo, Japan). Because the imaging intervals varied, the follow-up time was linearly scaled to a 0–1 range to adjust for temporal heterogeneity. This retrospective, anonymized study was conducted with ethics approval, and the requirement for informed consent was waived.

### Sample size and statistical considerations

Owing to the rarity of chronic empyema, this retrospective study included 45 patients (single-stage: *n* = 7; conventional: *n* = 38). Detecting between-group differences in the primary outcome of cavity reduction with 80% power required 12 patients per group (Cohen’s d = 0.7, per preliminary data). To reduce bias and strengthen inference, we applied propensity score matching, inverse probability of treatment weighting (IPTW), sensitivity analyses, and complementary predictive machine learning.

### Propensity score matching

To address selection bias, we performed 1:1 nearest-neighbor propensity score matching (no replacement, caliper 0.2) via logistic regression. Covariates included age, sex, body mass index (BMI), serum albumin, the neutrophil-to-lymphocyte ratio (NLR), preoperative cavity volume, performance status, resection type, decortication, BPF, and pathogens [10, 11]. Matching improved covariate balance.

### IPTW

To maximize the utilization of all case data, IPTW was implemented alongside propensity score matching [12, 13]. We prespecified the ATT as the primary estimand, targeting the effect of the single-stage approach among patients who actually received it. For ATT estimation, single-stage cases received weights of 1, and control cases were weighted by p/(1 − p), where p denotes the propensity score. For exploratory ATE estimation, treated and control cases were weighted by 1/p and 1/(1 − p), respectively. Because treatment assignment was strongly related to BPF (and all single-stage patients had BPF), overlap was limited; therefore, the ATE was interpreted as exploratory rather than a basis for generalization to the full cohort. Post-weight balance was assessed via standardized mean differences with sensitivity analyses at different trimming thresholds (1%, 5% and 10%) to evaluate the impact of extreme weights, confirming the robustness of the results. Treatment effects were estimated using propensity score–weighted linear regression with the cavity reduction ratio as the outcome, and 95% confidence intervals were computed using robust (sandwich) standard errors. A two-sided *p* < 0.05 was considered to indicate statistical significance.

### Machine learning models

Four ensemble models, CatBoost, XGBoost, random forest, and AdaBoost, were used as complementary predictive analyses to model cavity reduction and to explore potentially important predictors in a hypothesis-generating manner. These models were not intended to support causal inference or to estimate treatment effects. The features mirrored those used in the propensity score analysis. Stratified sampling with oversampling and undersampling was applied to mitigate rare-category imbalance in categorical predictors [14]. The hyperparameters were tuned via Optuna [15]. Five-fold cross-validation with 2,000 iterations of bootstrapping was used to assess performance. The evaluation metrics included R², RMSE, MAE, maximum error, and MAPE, with 95% confidence intervals (CIs). Feature contributions were assessed via Shapley additive explanations (SHAPs), a unified framework for explainable artificial intelligence, and stability scores were used to quantify robustness [16]. Python (ver. 3.8) with scikit-learn, XGBoost, CatBoost, SHAP, and Optuna libraries was used. A schematic illustration of the machine learning pipeline, including the five steps from preprocessing to model interpretation, is provided in Additional File 1: Figure S3. Given the small cohort size, we explicitly acknowledge the risk of overfitting and interpret ML findings as supportive and exploratory.

### Reporting guidance

This observational study is reported with reference to the STROBE guidance, and the predictive modeling components are described with reference to TRIPOD principles.

## Results

### Patient characteristics and propensity score matching

**Fig. 1:**
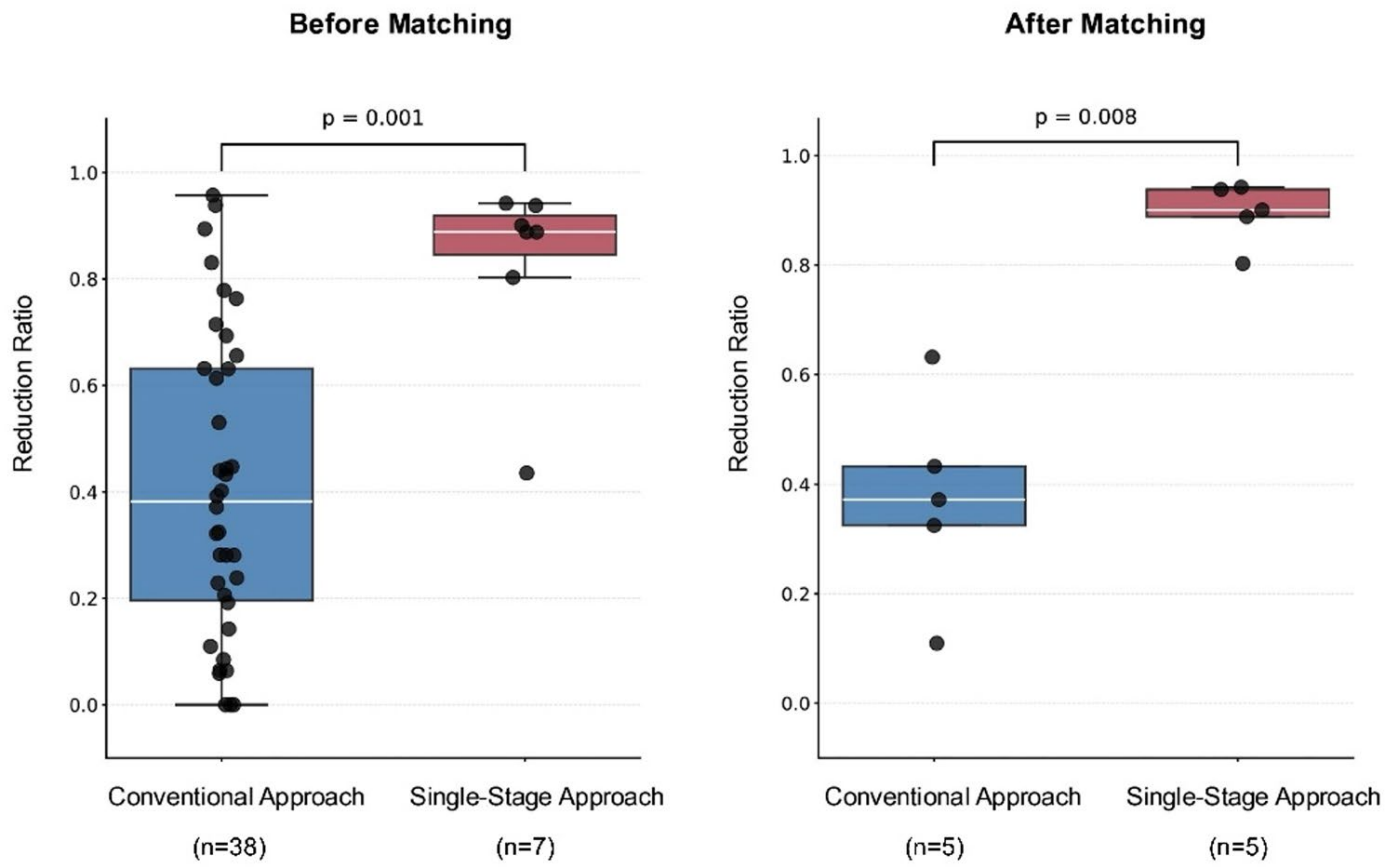
Empyema cavity reduction ratios with single-stage and conventional approaches. Box-and-whisker plots showing cavity reduction ratios in the single-stage and conventional groups before and after propensity score matching (unmatched: conventional *n* = 38, single-stage *n* = 7; matched: *n* = 5 per group). The single-stage group demonstrated significantly greater cavity reduction in both the unmatched and matched cohorts. P values were obtained using nonparametric tests (see Methods)

The baseline characteristics of the 45 patients (conventional: *n* = 38; single-stage: *n* = 7) were comparable (age: 68 vs. 74 years, albumin: 2.8 g/dL; NLR: 4.1 vs. 2.9; cavity volume: 294 vs. 297 mL), except for higher BPF (100% vs. 45%, *p* = 0.03), muscle flap (100% vs. 16%, *p* < 0.001), and NPWT use (100% vs. 21%, *p* < 0.001) in the single-stage approach (Table 1). Cavity reduction was significantly greater with the single-stage approach (89% vs. 38%, *p* = 0.001) and remained significant after matching (90% vs. 37%, *p* = 0.008) (Table 2; Fig. 1). In the matched cohort, the difference in medians for the cavity reduction ratio was approximately 50% points, indicating a large, clinically meaningful effect. Notably, because all single-stage patients had BPF, positivity/overlap across treatment groups was limited for this key clinical dimension, motivating ATT-focused interpretation in the weighted analyses.

**Table 1:** Patient characteristics and surgical outcomes of conventional and single-stage approaches in the management of chronic empyema

Characteristic	Total cohort (*n* = 45)	Conventional (*n* = 38)	Single-stage (*n* = 7)	*P* value	
Continuous variables, median (interquartile range)					
Age (years)	68 (65–74)	68 (66–74)	74 (62–78)	0.74	
Body mass index (kg/m^2^)	19.8 (17.8–22.5)	19.2 (17.3–22.3)	22.2 (21.8–22.5)	0.07	
Serum albumin (g/dL)	2.8 (2.3–3.2)	2.8 (2.3–3.2)	2.8 (2.7–3.3)	0.59	
Neutrophil-to-lymphocyte ratio	4.1 (2.2–6.9)	4.1 (2.3–7.2)	2.9 (2.1–6.3)	0.37	
Preoperative cavity volume (mL)	297 (167–526)	294 (177–584)	297 (76–403)	0.32	
Radiological follow-up interval (days)	79 (44–196)	80 (42–224)	75 (56–134)	0.91	
Time-normalized interval	0.04 (0.01–0.12)	0.04 (0.01–0.13)	0.04 (0.02–0.07)	0.91	
Reduction ratio (%)	44 (23–76)	38 (19–63)	89 (85–92)	0.001	
Categorical variables, n (%)					
Male sex	32 (71)	27 (71)	5 (71)	0.99	
Bronchopleural fistula	24 (53)	17 (45)	7 (100)	0.03	
Muscle flap transposition	13 (29)	6 (16)	7 (100)	< 0.001	
NPWT	15 (33)	8 (21)	7 (100)	< 0.001	
Performance status				0.21	
PS 0	17 (38)	14 (37)	3 (43)		
PS 1	19 (42)	17 (45)	2 (29)		
PS 2	1 (2)	0 (0)	1 (14)		
PS 3	1 (2)	1 (3)	0 (0)		
PS 4	4 (9)	3 (8)	1 (14)		
Missing	3 (7)	3 (8)	0 (0)		
Lung resection				0.03	
None	9 (20)	6 (16)	3 (43)		
Lobectomy/segmentectomy	13 (29)	9 (24)	4 (57)		
Partial resection	14 (31)	14 (37)	0 (0)		
Pneumonectomy	9 (20)	9 (24)	0 (0)		
Decortication				0.02	
Yes	14 (31)	9 (24)	5 (71)		
No	12 (27)	10 (26)	2 (29)		
Missing	19 (42)	19 (50)	0 (0)		
Pathogenic bacteria				0.53	
General bacteria	33 (73)	29 (76)	4 (57)		
Mycobacteria	7 (16)	5 (13)	2 (29)		
Fungi	5 (11)	4 (11)	1 (14)		

*NPWT*, negative pressure wound therapy; *PS*, performance status

**Table 2:** Propensity score-matched analysis of patient characteristics and surgical outcomes with conventional and single-stage approaches in the management of chronic empyema

Characteristic	Total cohort (*n* = 10)	Conventional (*n* = 5)	Single-stage (*n* = 5)	*P* value	
Continuous variables, median (interquartile range)					
Age (years)	66 (63–74)	65 (65–67)	74 (62–74)	0.92	
Body mass index (kg/m^2^)	22.1 (20.7–23.9)	20.4 (19.8–24.3)	22.3 (21.9–22.7)	0.55	
Serum albumin (g/dL)	2.9 (2.7–3.6)	2.9 (2.8–3.1)	2.9 (2.7–3.6)	0.99	
Neutrophil-to-lymphocyte ratio	6.0 (2.4–7.9)	6.3 (2.2–8.5)	5.7 (2.9–6.8)	0.69	
Preoperative cavity volume (mL)	181 (111–312)	243 (120–317)	118 (34–297)	0.55	
Radiological follow-up interval (days)	134 (42–211)	145 (33–233)	126 (65–141)	0.99	
Time-normalized interval	0.07 (0.01–0.13)	0.08 (0.01–0.14)	0.07 (0.03–0.08)	0.99	
Reduction ratio (%)	72 (39–89)	37 (32–43)	90 (89–94)	0.008	
Categorical variables, n (%)					
Male sex	5 (50)	2 (40)	3 (60)	0.99	
Bronchopleural fistula	10 (100)	5 (100)	5 (100)	0.99	
Muscle flap transposition	7 (70)	2 (40)	5 (100)	0.17	
NPWT	7 (70)	2 (40)	5 (100)	0.17	
Performance status				0.99	
PS 0	4 (40)	2 (40)	2 (40)		
PS 1	4 (40)	2 (40)	2 (40)		
PS 2	0 (0)	0 (0)	0 (0)		
PS 3	0 (0)	0 (0)	0 (0)		
PS 4	2 (20)	1 (20)	1 (20)		
Missing	0 (0)	0 (0)	0 (0)		
Lung resection				0.99	
None	3 (30)	1 (20)	2 (40)		
Lobectomy/segmentectomy	7 (70)	4 (80)	3 (60)		
Partial resection	0 (0)	0 (0)	0 (0)		
Pneumonectomy	0 (0)	0 (0)	0 (0)		
Decortication				0.51	
Yes	6 (60)	3 (60)	3 (60)		
No	3 (30)	1 (20)	2 (40)		
Missing	1 (10)	1 (20)	0 (0)		
Pathogenic bacteria				0.99	
General bacteria	6 (60)	3 (60)	3 (60)		
Mycobacteria	2 (20)	1 (20)	1 (20)		
Fungi	2 (20)	1 (20)	1 (20)		

*NPWT*, negative pressure wound therapy; *PS*, performance status

### Covariate balance assessment via propensity score weighting

ATT-based covariate balancing showed marked improvements in BMI (96.9% improvement), cavity volume (63.6%), NLR (55.5%), BPF (54.0%), NPWT (51.7%), and resection status (Table 3), whereas ATE yielded minor changes. ATT worsened the balance for sex (-4121.7%), albumin (-39.7%), and performance status (PS1) (-15.9%). Several covariates remained imperfectly balanced after weighting, particularly sex and serum albumin, suggesting the potential for residual confounding even after weighting; this should be considered when interpreting causal claims.

**Table 3:** Covariate balance assessment using standardized mean differences: comparison of ATT and ATE weighting

Variable	Unweighted SMD	ATT-Weighted SMD (% Change)	ATE-Weighted SMD (% Change)
Continuous variables			
Age (years)	0.18	0.11 (42.8)	0.15 (17.9)
Body mass index (kg/m^2^)	0.75	0.02 (96.9)	0.64 (15.5)
Serum albumin (g/dL)	0.19	0.27 (-39.7)	0.13 (34.4)
Neutrophil-to-lymphocyte ratio	0.40	0.18 (55.5)	0.34 (15.2)
Preoperative cavity volume (mL)	0.45	0.16 (63.6)	0.37 (17.9)
Time-normalized interval	0.26	0.16 (39.5)	0.25 (3.9)
Categorical variables			
Male Sex (vs. Female)	0.01	0.34 (-4121.7)	0.01 (11.8)
Bronchopleural fistula (vs. absent)	1.70	0.49 (54.0)	1.04 (3.2)
Muscle flap transposition (vs. not performed)	2.46	1.70 (30.8)	2.41 (1.8)
NPWT (vs. not applied)	2.06	0.99 (51.7)	1.96 (5.0)
Performance status (vs. PS0)			
PS1	0.32	0.37 (-15.9)	0.37 (-14.7)
PS2	1.01	1.01 (0.0)	1.23 (-21.2)
PS3	0.18	0.20 (-13.4)	0.18 (-2.2)
PS4	0.22	0.07 (68.5)	0.26 (-16.3)
Lung resection (vs. not performed)			
Lobectomy/segmentectomy	0.75	0.21 (72.6)	0.79 (-5.3)
Partial resection	0.59	0.28 (52.6)	0.58 (1.9)
Pneumonectomy	0.81	0.39 (52.2)	0.79 (2.3)
Decortication (vs. no)	0.85	0.79 (7.1)	0.93 (-9.8)
Pathogenic bacteria (vs. Fungi)			
General bacteria	0.42	0.36 (14.9)	0.32 (25.2)
Mycobacteria	0.12	0.27 (-128.8)	0.09 (23.5)

*ATE*, average treatment effect; *ATT*, average treatment effect on the treated; *NPWT*, negative pressure wound therapy; *PS*, performance status; *SMD*, standardized mean difference. % Change indicates improvement (positive values) or worsening (negative values) in balance after weighting. For covariates with near-zero unweighted SMD, the percent change can be unstable and should be interpreted cautiously

### Treatment effect estimation via propensity score weighting

**Fig. 2:**
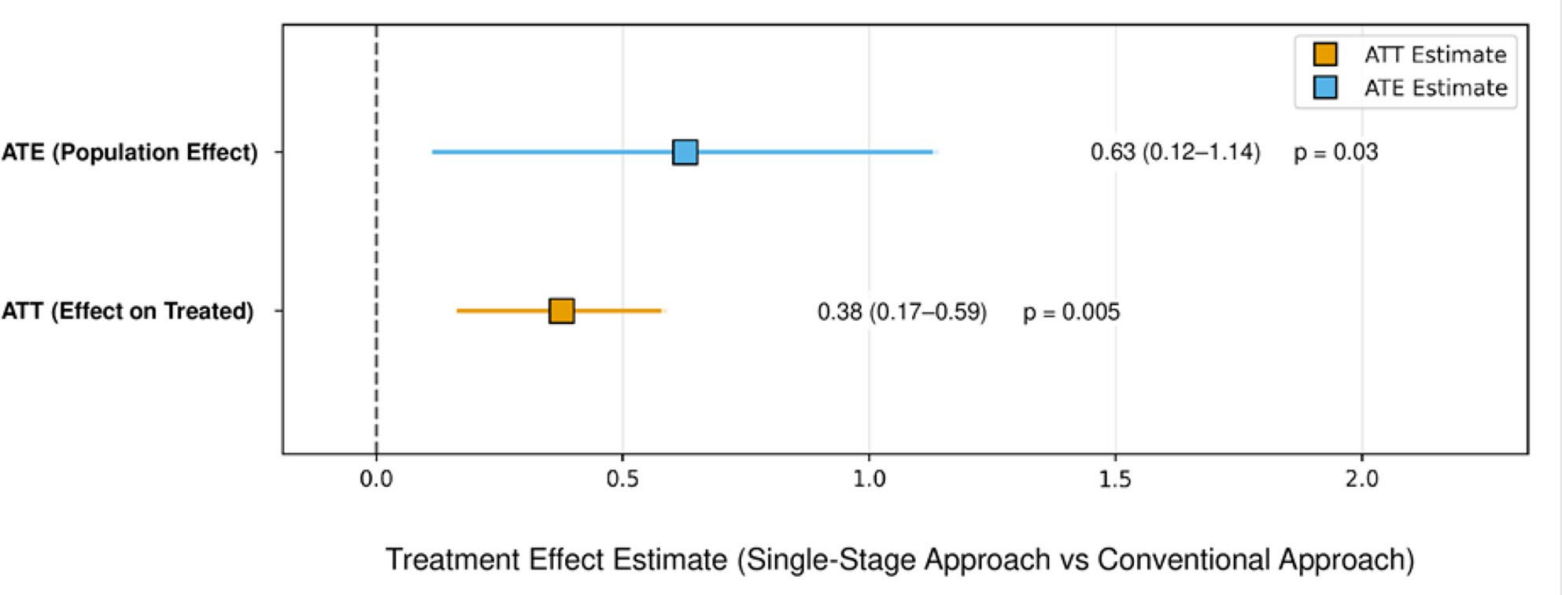
Treatment effect estimates via different propensity score weighting methods. Forest plot comparing IPTW estimates of the single-stage approach versus the conventional approach. The ATT estimate (effect among treated patients) is the prespecified primary estimand, whereas the ATE is presented as exploratory given limited overlap related to bronchopleural fistula and treatment assignment

Propensity score-weighted regression demonstrated significant effects in favor of the single-stage approach. Consistent with our prespecified primary estimand, the ATT model estimated an effect size of 0.38 (95% CI: 0.17–0.59, *p* = 0.005) (Table 4; Fig. 2). The ATE model estimated an effect size of 0.63 (95% CI: 0.12–1.14, *p* = 0.03) but was interpreted as exploratory given limited overlap driven by the strong relationship between BPF and treatment assignment. The model fit was robust in both cases (ATT: R²=0.65, adjusted R²=0.59; ATE: R²=0.58, adjusted R²=0.51). Additional File 2: Table S1 summarizes the regression coefficients from the ATT and ATE models, highlighting the treatment approach as the strongest predictor. Sensitivity analyses across trimming thresholds (1%, 5% and 10%) demonstrated consistent estimates: ATT (0.377, 0.383, and 0.384) and ATE (0.627, 0.625, and 0.625), supporting the robustness to extreme weights (Additional File 1: Figure S4).

**Table 4:** Comparison of treatment effects using different propensity score weighting methods

Analysis Type	Treatment Effect (95% CI)	*P* value	*R* ^2^	Adjusted *R*^2^
ATT (Single-stage vs. conventional)	0.38 (0.17–0.59)	0.005	0.65	0.59
ATE (Single-stage vs. conventional)	0.63 (0.12–1.14)	0.03	0.58	0.51

*ATE*, average treatment effect; *ATT*, average treatment effect on the treated; *CI*, confidence interval; *R*^*2*^, coefficient of determination

### Model optimization and performance of machine learning models

Hyperparameter optimization for machine learning models: As complementary predictive analyses, we trained four models (CatBoost, XGBoost, random forest, and AdaBoost) using clinical features. The parameters were optimized via Optuna (Additional File 2: Table S2): CatBoost (443 iterations, 0.0542 learning rate, depth 4), XGBoost (292 estimators, max depth 3), random forest (159 estimators, max depth 7), and AdaBoost (60 estimators, 0.7163 learning rate). CatBoost performed best (Additional File 2: Table S3): R²=0.72 (95% CI: 0.41–0.87), RMSE = 0.20, MAE = 0.09, MAPE = 32.3, and was selected for subsequent interpretation.

### Prediction performance evaluation of the CatBoost model

Additional File 1: Figure S5 shows the strong prediction accuracy. Predictions in the single-stage approach clustered at 0.8–1.0. Those in the conventional approach range widely.

### Assessment of feature importance in cavity reduction prediction

**Fig. 3:**
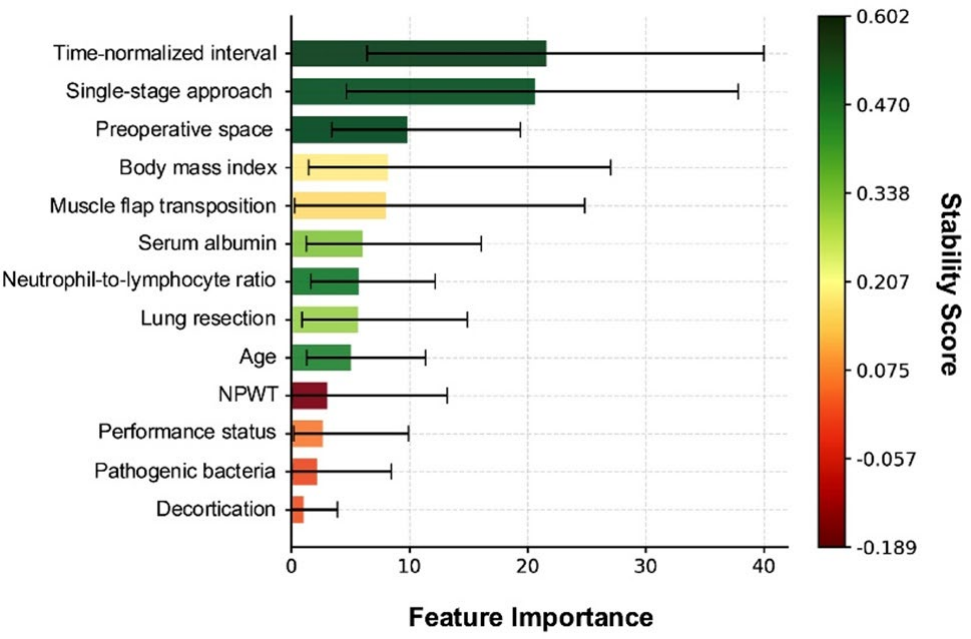
Feature importance analysis for empyema cavity reduction prediction. The horizontal bars represent the relative importance of the clinical and treatment variables for predicting cavity reduction, with 95% confidence intervals. The color gradient indicates the stability scores. These importance measures are predictive and hypothesis-generating and should not be interpreted as causal effects

The time-normalized interval, treatment approach, and preoperative cavity volume were the top predictors (Fig. 3), with BMI, muscle flap, and albumin being moderately important. NPWT alone, PS, pathogens, and decortication had less impact.

## Discussion

We compared a single-stage approach (OWT with simultaneous muscle flap transposition and early NPWT) with conventional staged management for chronic empyema using 3D volumetric outcomes. Across PSM and weighted analyses, the single-stage strategy was consistently associated with greater cavity reduction. In addition, complementary predictive machine learning analyses identified treatment approach and time-related variables as important predictors, supporting the predictive coherence of the overall signal across analytic frameworks.

For causal interpretation, we prespecified the ATT as the primary estimand, which targets the effect of the single-stage approach among patients who received it. This focus is clinically relevant because the single-stage strategy was preferentially applied to more complex cases, particularly those with bronchopleural fistula. In contrast, generalization to a broader population-level effect (ATE) requires stronger positivity/overlap; because BPF was strongly associated with treatment assignment and all single-stage patients had BPF, overlap was limited, and the ATE should be viewed as exploratory.　 Under limited overlap, the ATE can rely on extrapolation beyond the region of common support and may therefore be unstable and less interpretable for broad generalization.

Although ATT weighting improved balance for several clinically important covariates, imbalance persisted (and in some instances worsened) for sex and serum albumin, raising the possibility of residual confounding. Serum albumin is plausibly related to wound healing and recovery; therefore, incomplete balance could influence effect estimates. These limitations reinforce the need to interpret results primarily in an ATT framework and to validate findings in larger multicenter cohorts.

Despite the widespread use of stepwise management with staged infection control and cavity obliteration [17–19], chronic empyema remains clinically challenging, particularly when persistent infection and pleural fibrosis limit lung re-expansion and durable cavity closure.　 Pathophysiologically, prolonged infection promotes fibroblast activation, matrix deposition, and pleural rigidity [20–22], thereby limiting lung expansion and cavity resolution. The single-stage approach may achieve earlier cavity closure via synergistic effects: OWT for drainage, muscle flap for obliteration and BPF closure, and NPWT for infection control and re-expansion.

In our cohort, the single-stage approach was applied exclusively to patients with BPF, reflecting real-world surgical decision-making for complex empyema. Despite all single-stage patients with BPF being typically refractory [23], cavity reduction was large; however, confounding by indication remains possible because treatment assignment was not randomized. These findings therefore support the feasibility and potential value of the single-stage strategy in difficult BPF-associated empyema, while underscoring the need for prospective validation and clearer selection criteria.

From a practical standpoint, candidates for a single-stage strategy may include patients with chronic empyema complicated by BPF, those with anatomy suitable for reliable muscle flap coverage, and those with preserved functional reserve (PS 0–2) in whom early cavity obliteration is desirable. Conversely, a staged strategy may be preferable for patients with poor functional reserve (PS 3–4), profound malnutrition or severe hypoalbuminemia (e.g., < 2.0 g/dL), uncontrolled sepsis, extensive necrosis, or inadequate muscle mass/vascularity for a durable flap. In all cases, multidisciplinary assessment and individualized surgical planning remain essential.

The SHAP analysis highlighted the time-normalized interval as a dominant predictor. This likely reflects both biological healing dynamics (progressive cavity contraction and granulation over time) and study-design factors (heterogeneous timing of follow-up imaging that may correlate with clinical course and decision-making). Although we normalized follow-up intervals to reduce temporal heterogeneity, residual time-related effects may persist. Future prospective studies should incorporate standardized imaging time points to better disentangle temporal dynamics from treatment-related effects.

This study has several limitations. The small single-stage sample (*n* = 7) constrains precision and increases susceptibility to residual confounding, despite the use of propensity score matching and IPTW. Accordingly, our causal interpretation is centered on the ATT, whereas the ATE should be considered exploratory due to limited overlap. The retrospective single-center design and the exclusive inclusion of BPF patients in the single-stage group may limit generalizability. The follow-up timing varied among patients. Although the intervals were normalized, unmeasured time-related factors may have persisted. To minimize bias in this small-sample setting, we intentionally focused our inference on the imaging-based primary endpoint and did not analyze secondary clinical outcomes. Long-term clinical outcomes (such as durability of closure and patient-reported quality of life) were not assessed. Finally, while the machine learning analyses yielded consistent patterns, the limited sample size increased the risk of overfitting; thus, machine learning results should be interpreted as predictive and hypothesis-generating rather than causal. Prospective multicenter validation is warranted.

## Conclusions

This study suggests that a single-stage approach combining OWT, muscle flap transposition, and early NPWT is associated with greater empyema cavity reduction than conventional management in patients treated with this strategy, including those with BPF. Causal interpretation is most appropriate for the ATT, whereas the ATE should be considered exploratory due to limited overlap. Consistent findings across complementary analyses support prospective multicenter validation and clearer clinical criteria for patient selection.

## Supplementary Information

Below is the link to the electronic supplementary material.

Supplementary Material 1: Figures S1–S5. Graphical abstract; intraoperative/NPWT steps; ML workflow; trimming sensitivity plots; model prediction scatter.

Supplementary Material 2: Tables S1–S3. Weighted regression coefficients (ATT/ATE); ML hyperparameters; model performance metrics.

## Data Availability

The datasets and analysis codes used and/or analyzed during the current study are available from the corresponding author upon reasonable request and are subject to patient privacy restrictions.

## Abbreviations

3D: Three-dimensional
3D CT: Three-dimensional computed tomography
ATE: Average treatment effect
ATT: Average treatment effect on the treated
BMI: Body mass index
BPF: Bronchopleural fistula
CI: Confidence interval
CT: Computed tomography
IPTW: Inverse probability of treatment weighting
MAE: Mean absolute error
MAPE: Mean absolute percentage error
NLR: Neutrophil-to-lymphocyte ratio
NPWT: Negative pressure wound therapy
OWT: Open window thoracostomy
PS: Performance status
R²: Coefficient of determination
RMSE: Root-mean-square error
SHAP: Shapley additive explanations
STROBE: Strengthening the reporting of observational studies in epidemiology
TRIPOD: Transparent reporting of a multivariable prediction model for individual prognosis or diagnosis
VAC: Vacuum-assisted closure
