# Supplementary material for: Single-stage open window thoracostomy with simultaneous muscle flap transposition and early negative pressure wound therapy for chronic empyema: a propensity score- and machine learning-based study
Source: J Cardiothorac Surg. 2026 Apr 3;21:235. doi: 10.1186/s13019-026-03997-y (PMC13173702; doi:10.1186/s13019-026-03997-y)
Supplement: Supplementary file 1 — Supplementary Material 1: Figures S1–S5. Graphical abstract; intraoperative/NPWT steps; ML workflow; trimming sensitivity plots; model prediction scatter. [file 13019_2026_3997_MOESM1_ESM.docx]

**Supplementary Figures**

**Figure S1. Graphical abstract summarizing study design and key findings.**

The single-stage approach combining open window thoracostomy, muscle flap transposition, and early NPWT achieved superior cavity reduction relative to the conventional stepwise method. NPWT, negative pressure wound therapy

**
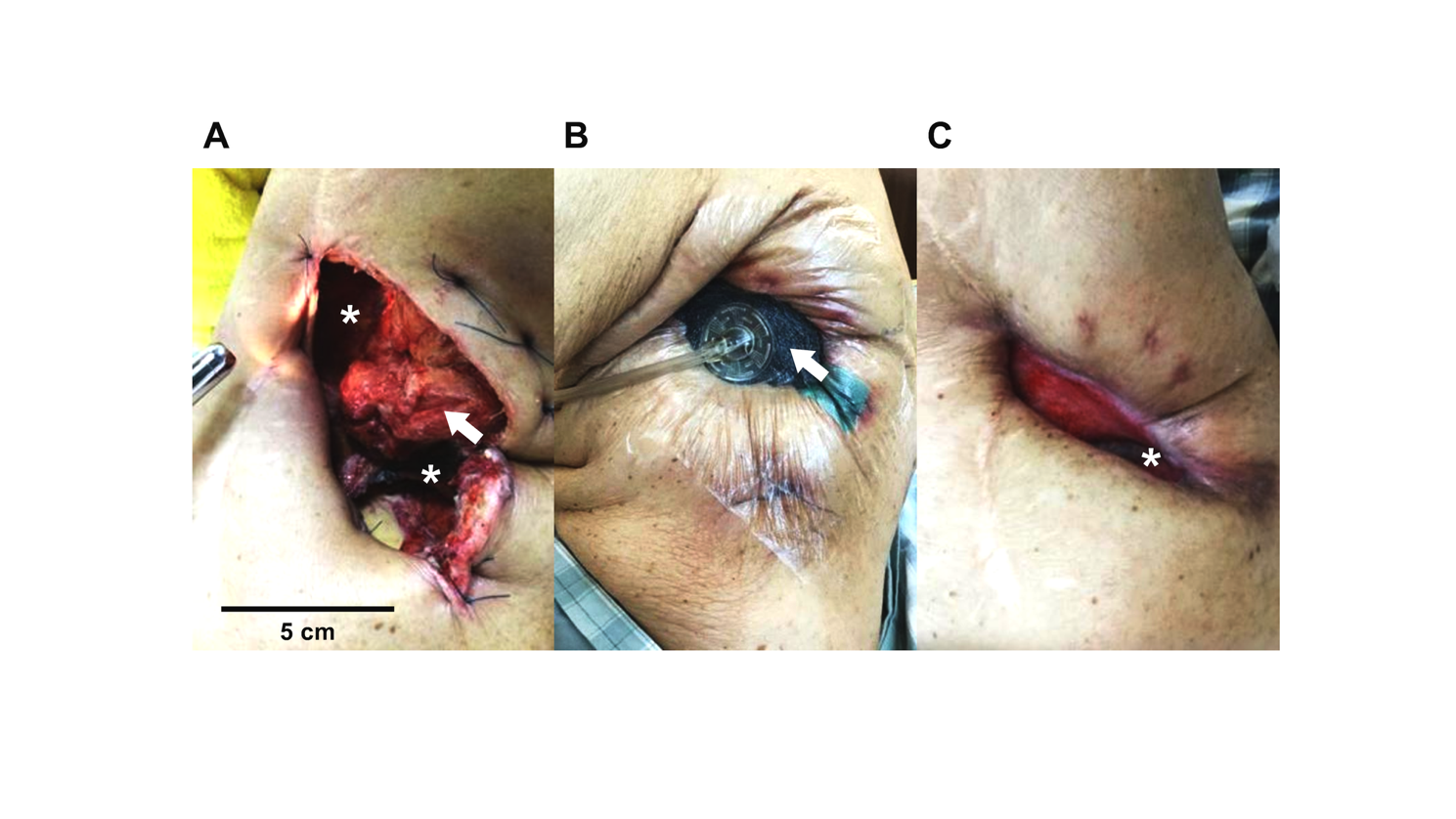
**

**Figure S2. Stages of the single-stage approach for management of empyema space.** (A) Intraoperative view after open window thoracostomy with simultaneous muscle flap transposition (asterisk, empyema cavity; white arrow, latissimus dorsi flap). (B) Implementation of negative pressure wound therapy within 72 hours (white arrow, VAC system). (C) Healed wound after therapy (asterisk, reduced empyema cavity). VAC, vacuum-assisted closure

**
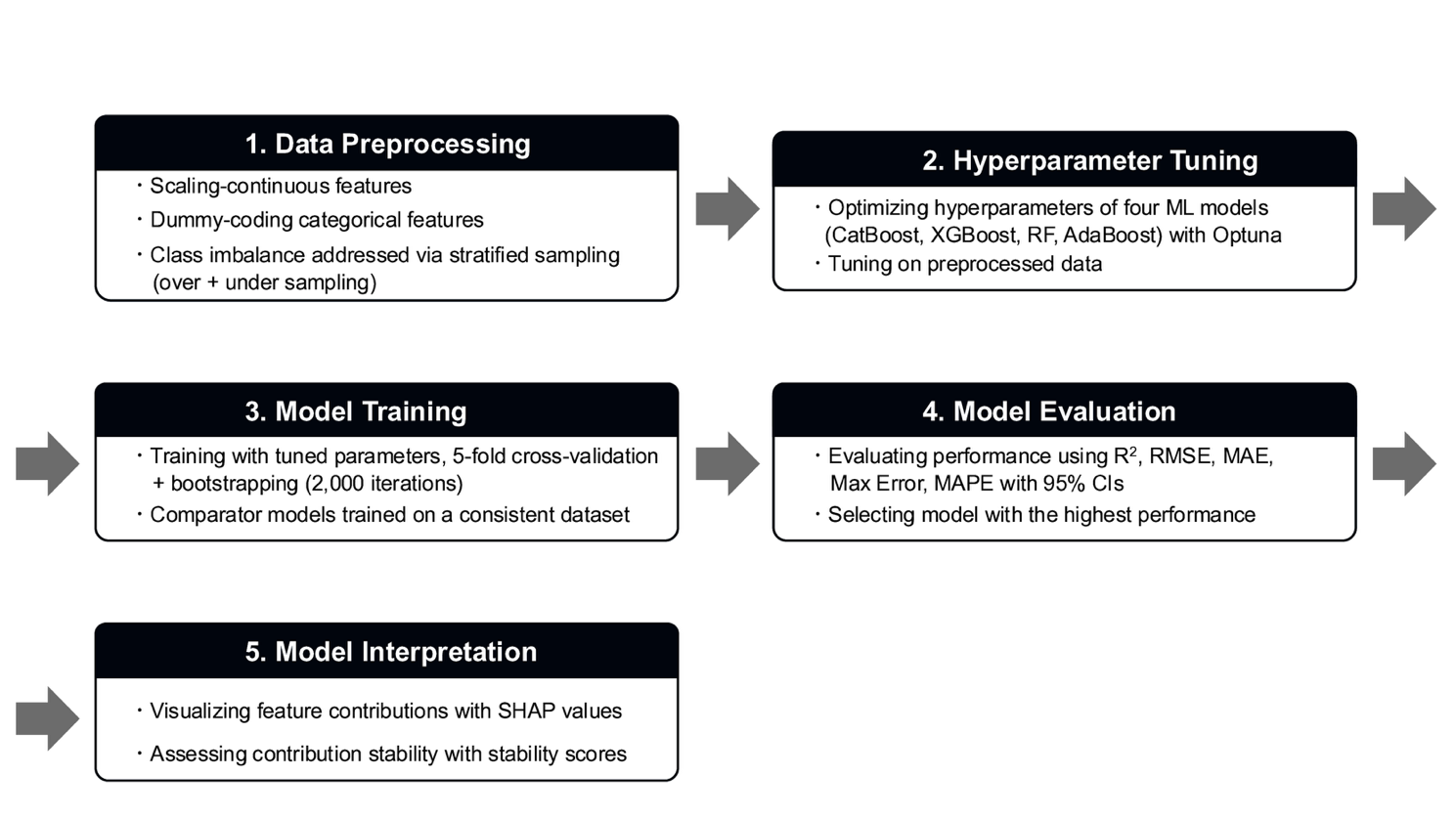
**

**Figure S3. Schematic representation of the five-step machine learning pipeline used in this study.**

(1) Data preprocessing with scaling and dummy-coding, and addressing rare-category imbalance in categorical predictors; (2) hyperparameter tuning using Optuna for four machine learning models (CatBoost, XGBoost, Random Forest, AdaBoost); (3) model training using 5-fold cross-validation and 2,000 bootstrap iterations; (4) model evaluation using five metrics (R², RMSE, MAE, maximum error, MAPE); and (5) model interpretation with SHAP values and stability scores. MAE, mean absolute error; MAPE, mean absolute percentage error; RMSE, root-mean-square error; R^2^, coefficient of determination; SHAP, Shapley additive explanations


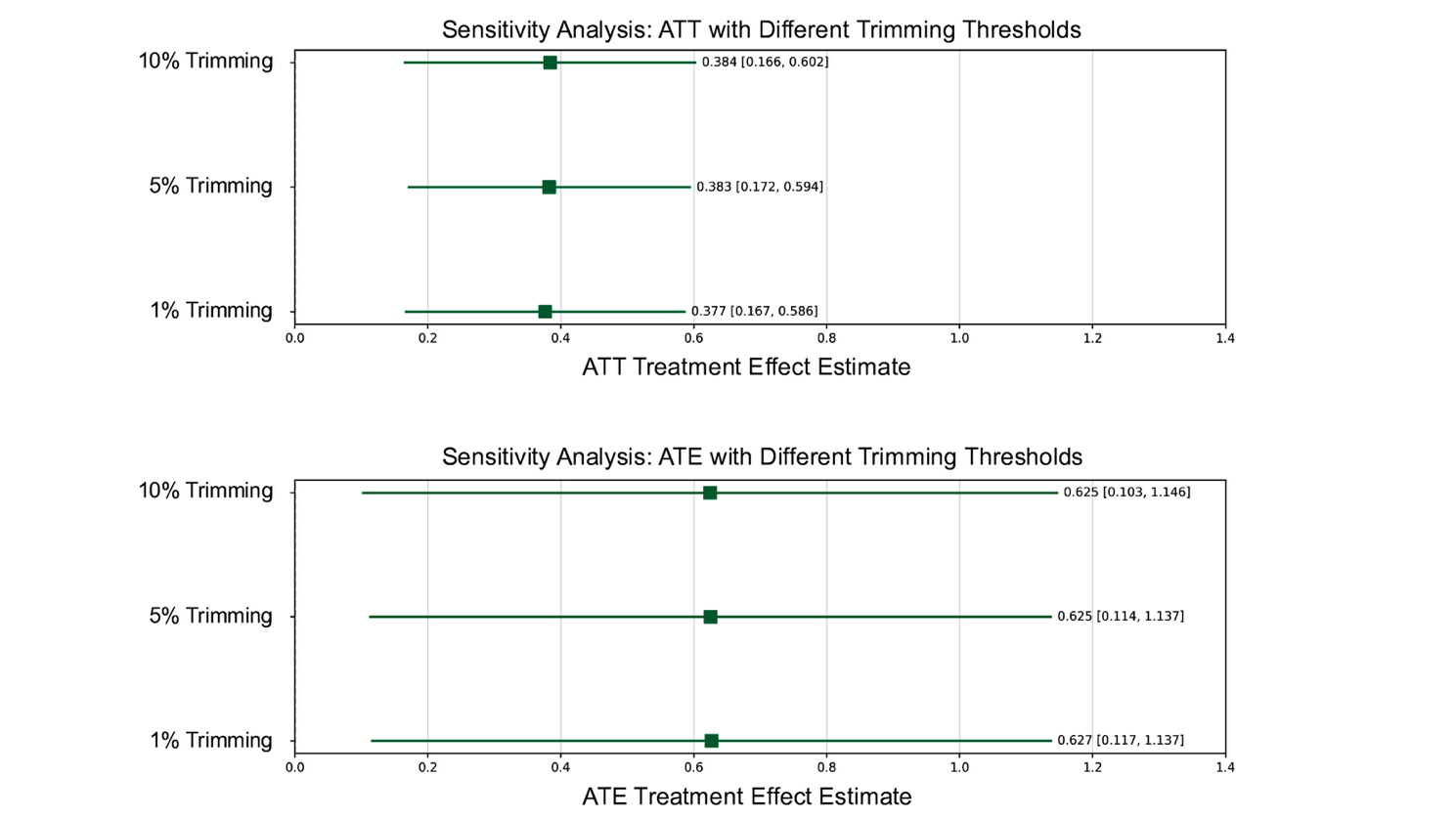


**Figure S4. Sensitivity analysis of treatment effect estimates.**

Forest plots demonstrating consistency of treatment effect estimates across multiple trimming thresholds (1%, 5%, and 10%) for both ATT (upper panel) and ATE (lower panel) analyses. ATE, average treatment effect; ATT, average treatment effect on the treated


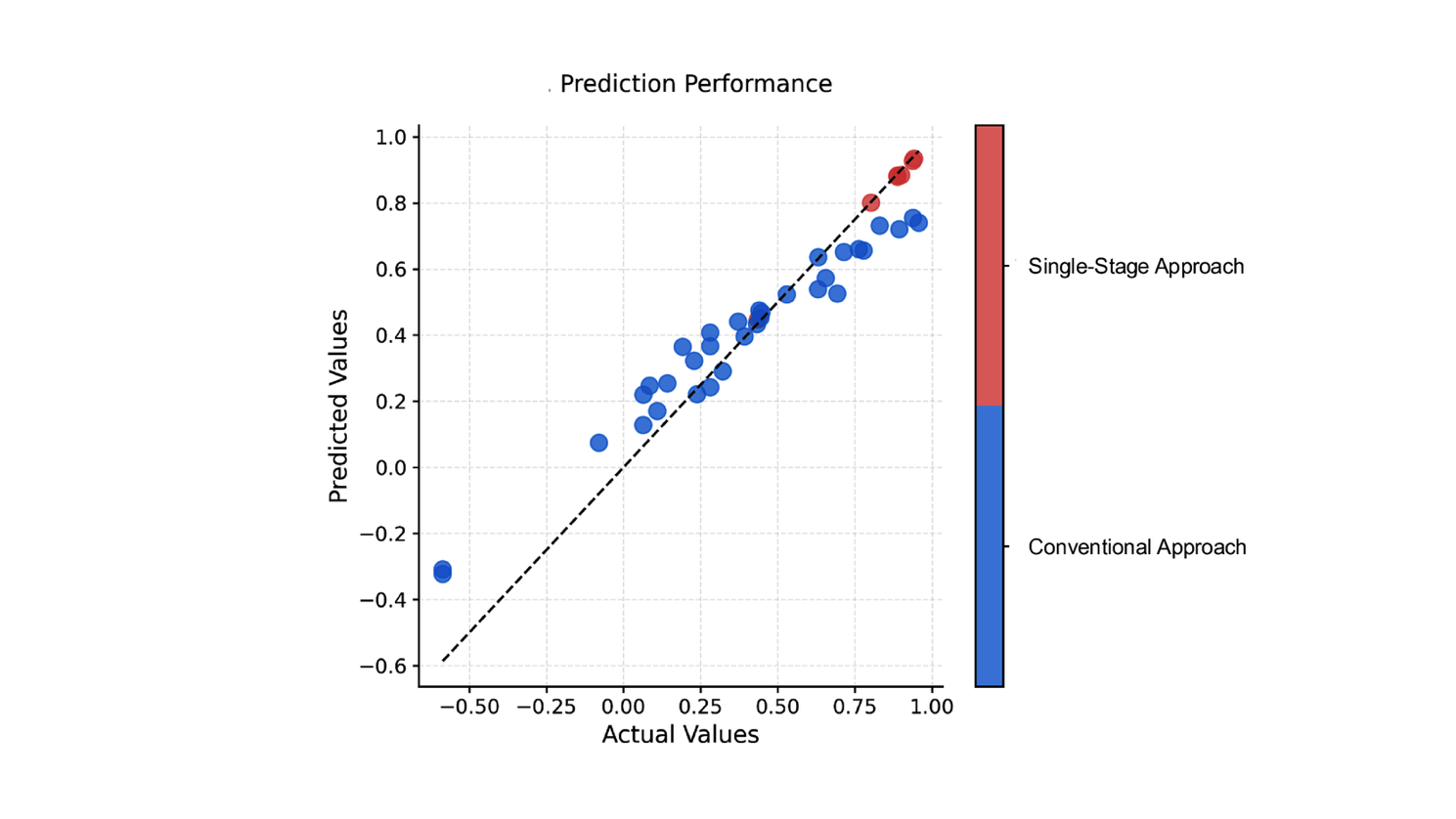


**Figure S5. Prediction performance of the CatBoost machine learning model.**

Scatter plot illustrating the correlation between predicted and actual cavity reduction values, with single-stage cases (red) clustering in the high reduction range and conventional cases (blue) showing a wider distribution.
