# Supplementary material for: Single-stage open window thoracostomy with simultaneous muscle flap transposition and early negative pressure wound therapy for chronic empyema: a propensity score- and machine learning-based study
Source: J Cardiothorac Surg. 2026 Apr 3;21:235. doi: 10.1186/s13019-026-03997-y (PMC13173702; doi:10.1186/s13019-026-03997-y)
Supplement: Supplementary file 2 — Supplementary Material 2: Tables S1–S3. Weighted regression coefficients (ATT/ATE); ML hyperparameters; model performance metrics. [file 13019_2026_3997_MOESM2_ESM.docx]

**Supplementary Tables**

**Table S1. Comprehensive Results of the Propensity Score Weighted Regression Models**

| Variable | ATT Coefficient  (95% CI) | p | ATE Coefficient  (95% CI) | p |
| --- | --- | --- | --- | --- |
| Intercept | -2.92 (-5.60, -0.24) | 0.04 | -0.95 (-4.41, 2.51) | 0.51 |
| Treatment Approach | | | | |
| Single-Stage (vs. Conventional) | 0.38 (0.17, 0.59) | 0.005 | 0.63 (0.12, 1.14) | 0.03 |
| Continuous variables | | | | |
| Age, years | 0.02 (0.004, 0.033) | 0.02 | 0.01 (-0.02, 0.04) | 0.38 |
| Body mass index, kg/m^2^ | 0.03 (-0.02, 0.07) | 0.18 | 0.01 (-0.05, 0.07) | 0.64 |
| Serum albumin, g/dL | 0.43 (0.11, 0.76) | 0.02 | 0.24 (-0.39, 0.86) | 0.38 |
| Neutrophil-to-lymphocyte ratio | 0.05 (0.02, 0.07) | 0.004 | 0.01 (-0.03, 0.06) | 0.49 |
| Preoperative cavity volume, mL | 0.001 (0.000, 0.001) | 0.01 | 0.000 (-0.000, 0.001) | 0.71 |
| Time-normalized interval | -0.32 (-2.46, 1.82) | 0.72 | -0.50 (-2.39, 1.39) | 0.53 |
| Categorical variables | | | | |
| Male sex (vs. Female) | 0.76 (0.54, 0.97) | <0.001 | 0.45 (-0.04, 0.94) | 0.06 |
| Bronchopleural fistula (vs. absent) | -0.06 (-0.29, 0.17) | 0.53 | -0.22 (-0.68, 0.24) | 0.27 |
| Muscle flap transposition (vs. not performed) | -0.21 (-0.39, -0.03) | 0.03 | -0.16 (-0.65, 0.32) | 0.43 |
| NPWT (vs. not applied) | 0.48 (0.12, 0.85) | 0.02 | -0.01 (-0.76, 0.73) | 0.96 |
| Performance status (vs. PS0) |  |  |  |  |
| PS1 | -0.79 (-1.01, -0.53) | 0.001 | -0.33 (-0.74, 0.08) | 0.10 |
| PS2 | 0.32 (-0.09, 0.72) | 0.10 | -0.01 (-0.73, 0.71) | 0.98 |
| PS3 | 0.000 (-0.000, 0.000) | 0.28 | -0.000 (-0.000, 0.000) | 0.67 |
| PS4 | -0.65 (-0.88, -0.43) | 0.001 | -0.47 (-1.03, 0.08) | 0.08 |
| Lung resection (vs. not performed) |  |  |  |  |
| Lobectomy/Segmentectomy | 0.24 (-0.04, 0.52) | 0.08 | 0.27 (-0.20, 0.74) | 0.20 |
| Partial resection | 0.51 (0.13, 0.88) | 0.02 | 0.173 (-0.34, 0.69) | 0.43 |
| Pneumonectomy | 0.54 (-0.80, 1.88) | 0.35 | 0.084 (-0.972, 1.140) | 0.85 |
| Decortication (vs. no) | -0.51 (-0.69, -0.32) | 0.001 | -0.19 (-0.75, 0.37) | 0.43 |
| Pathogenic bacteria (vs. Fungi) |  |  |  |  |
| General bacteria | -0.27 (-0.57, 0.03) | 0.07 | -0.45 (-1.23, 0.32) | 0.19 |
| Mycobacteria | 0.69 (0.16, 1.13) | <0.001 | 0.45 (-0.04, 0.94) | 0.06 |

*ATE*, average treatment effect; *ATT*, average treatment effect on the treated; *NPWT*, negative pressure wound therapy; *PS*, performance status. ^*^The outcome variable was reduction ratio

**Table S2. Machine Learning Model Hyperparameters for Reduction Ratio Prediction**

| Hyperparameters for CatBoost | |
| --- | --- |
| iterations | 443 |
| learning_rate | 0.0542 |
| depth | 4 |
| l2_leaf_reg | 0.0365 |
| bagging_temperature | 0.2732 |
| random_strength | 0.2641 |
| min_data_in_leaf | 20 |
| Leaf_estimation_iterations | 7 |

| Hyperparameters for XGBoost | |
| --- | --- |
| n_estimators | 292 |
| max_depth | 3 |
| learning_rate | 0.0575 |
| min_child_weight | 4 |
| subsample | 0.8667 |
| colsample_bytree | 0.8389 |
| gamma | 0.0004 |

| Hyperparameters for random forest | |
| --- | --- |
| n_estimators | 159 |
| max_depth | 7 |
| min_samples_split | 3 |
| min_samples_leaf | 1 |
| max_features | 0.4715 |

| Hyperparameters for AdaBoost | |
| --- | --- |
| n_estimators | 60 |
| learning_rate | 0.7163 |
| loss | linear |

**Table S3. Performance Metrics of Machine Learning Models for Predicting Reduction Ratio**

| Machine learning  model | Assessment metrics (95% CI) | | | | |
| --- | --- | --- | --- | --- | --- |
|  | **R^2^** | **RMSE** | **MAE** | **Max error** | **MAPE** |
| CatBoost | 0.72 (0.41–0.87) | 0.20 (0.14–0.29) | 0.09 (0.06–0.12) | 0.73 (0.47–1.16) | 32.3 (12.7–61.8) |
| XGBoost | 0.69 (0.36–0.85) | 0.21 (0.15–0.30) | 0.10 (0.07–0.13) | 0.71 (0.47–1.22) | 35.2 (17.9–59.8) |
| random forest | 0.64 (0.41–0.77) | 0.22 (0.18–0.29) | 0.15 (0.13–0.18) | 0.73 (0.42–1.10) | 55.7 (39.3–77.7) |
| AdaBoost | 0.64 (0.32–0.84) | 0.22 (0.15–0.32) | 0.13 (0.10–0.17) | 0.80 (0.45–1.29) | 50.9 (31.7–81.4) |

*CatBoost*, categorical boosting; *CI*, confidence interval; *MAE*, mean absolute error; *MAPE*, mean absolute percentage error; *Max error*, maximum prediction error; *R^2^*, coefficient of determination; *RMSE*, root-mean-square error; *XGBoost*, extreme gradient boosting.
